# Editorial: Development, Wellbeing, and Lifelong Learning in Individuals With a Dual Sensory Loss

**DOI:** 10.3389/fpsyg.2021.790549

**Published:** 2021-12-09

**Authors:** Marleen J. Janssen, Timothy S. Hartshorne, Walter Wittich

**Affiliations:** ^1^Department of Inclusive and Special Needs Education, Institute for Deafblindness, University of Groningen, Groningen, Netherlands; ^2^Department of Psychology, Central Michigan University, Mount Pleasant, MI, United States; ^3^School of Optometry, Université de Montréal, Montreal, QC, Canada

**Keywords:** deafblindness, dual sensory impairment, education, rehabilitation, low vision, hard-of-hearing

Dual sensory loss, also known as deafblindness, affects individuals at every developmental stage and age. Individuals affected by the impairment of both vision and hearing represent an extremely vulnerable group. Their unique combination of sensory disabilities has a major impact on their access to information, communication, social networks as well as orientation and mobility (McInnes, 1999; Rødbroe and Janssen, 2006; Dammeyer, 2012; Hersh, 2013; Ask Larsen and Damen, 2014; Vervloed and Damen, 2016; Theil et al., 2020).

Although “dual sensory loss” is recognized as a unique disability (European Parliament, 2004), meaning that everyone with deafblindness has the right to specifically tailored education and rehabilitation, policies and practices are nevertheless fragmented. Research on deafblindness is still lacking. This Research Topic, with a collection of the latest original research findings, will serve to bridge the gap between theory and practice thereby strengthening professionals' knowledge, institutions, and systems in their mission to support wellbeing, development, and lifelong learning of people with dual sensory loss. The current fragmentation of policies and practices is the result of several reasons. Significantly, dual sensory deprived individuals can be divided into three subgroups with their own focus areas. Classification is mainly attributable to the onset of the dual sensory loss: (a) Congenital deafblindness: from birth; (b) Acquired deafblindness: after language acquisition (during infancy, puberty or young adolescence); and (c) Age-related deafblindness: affecting individuals over 50, due to aging (Jaiswal et al., 2018); or as a result of a syndrome. Many syndromes can lead to deafblindness, CHARGE syndrome being the most common for congenital deafblindness, and different types of USHER syndrome for acquired deafblindness (Regenbogen and Coscas, 1985). The goal of this Research Topic was to contribute to the “cutting edge nature” of research on dual sensory loss and reunite the three fields within deafblind education and rehabilitation. Each of the 20 papers that are included have contributed toward meeting this goal, covering themes which can be divided in four main clusters, namely (1) wellbeing and health; (2) communication, learning, and access to information; (3) importance of assistive technology; and (4) research, professionalization and support. In addition to papers reporting original research, this Research Topic also includes several review and theory articles, giving us new empirical evidence and innovative ideas for practical approaches.

## Wellbeing and Health

The first chapter in this Research Topic focuses on the wellbeing and health of individuals with a dual sensory loss. In this chapter the three subgroups are addressed, and even broadened with the subgroup of people with combined deafblindness and intellectual disabilities. The two studies by De Vaan et al. focus on wellbeing and stress in individuals with combined intellectual and sensory disabilities. De Vaan, Vervloed, et al. investigated stress affect regulation and attachment behaviors in 60 children and adults with combined sensory and intellectual disabilities of which 16 participants were deafblind. The researchers concluded based on specific questionnaires filled in by caregivers, that almost all participants showed signs of disturbed attachment. Having an additional diagnosis of Autism Spectrum Disorder (ASD) resulted in more disturbed attachment, and additional manic and hyperactive behavior and social avoidance. In the other study, De Vaan, Beijers, et al. found no differences in stress levels between those with and without ASD, however cortisol levels were correlated with stereotyped and repetitive behaviors which makes it likely that these behaviors are stress related.

Wanka studied the development of identity in a very interesting case study with Janosch, a young man of 23 years old with CHARGE Syndrome utilizing the CLET technique (Van Schalkwyk, Collage Life Story Elicitation). This technique had been earlier evaluated in adults with Acquired deafblindness in a study by Van de Molengraft (2011). Wanka adapted this procedure for 13 participants with CHARGE Syndrome, among which Janosch was the most representative. This technique makes use of a dialogical protocol to support the individual in creating his own life story based on the several voices in the individual himself. The findings indicated that Janosch did not associate his core identity with CHARGE syndrome, but gives more relevance to other areas of significance in his life, such as animals and to the community to which he belongs. It is clear that these aspects contribute enormously to his wellbeing.

Wahlqvist contributed two methodologically very different studies focused on Health issues. In the first study, Wahlqvist, Möller, et al. used a large database from the Swedish Usher Database to compare three groups of people with Usher Syndrome, namely type 1, type 2 and type 3, on similarities and differences in health, social trust, and financial situation. The data of 162 people with Usher were analyzed and the results demonstrated more similarities than differences between the three groups. It was recommended that more evidence is needed to confirm findings from clinical settings and the life stories told by people with Usher Syndrome. In the other study, Wahlqvist, Bjork, et al. studied Health-related Quality of Life, Family Climate, and Sense of Coherence with 14 Parents with acquired deafblindness, 6 partners, and 18 children. The parents reported the poorest Health-related Quality of Life. A moderate to low Sense of Coherence (comprehensibility, manageability and meaningfulness) was reported by all family members. The Family Climate questionnaire revealed a positive aspect in the closeness across the family members, but negative aspects, mainly reported by the parents, in the chaos and expressiveness in the families. It becomes clear that deafblindness not only affects the individual but the whole family, which has important implications for finding appropriate family support.

Vreeken et al. performed a short intervention of 3–5 weeks with 131 older adults (>50 years) with low vision and self-reported hearing disability, and their communication partners (*N* = 113) based on the Dual Sensory Loss Protocol. They used a randomized control trial design, with an intervention group and a waiting-list control group, which is very unique for this target population. It was concluded that the DSL-protocol did not clearly contribute to the improvement of wellbeing and communication. Adaptation of the protocol and involvement of mental health care professionals was recommended.

## Communication, Learning, and Access to Information

In Chapter 2, five studies are reported on individuals with congenital deafblindness. Two studies address access to information for people with acquired deafblindness. Three studies on communication mainly explore strategies and competences of communication partners. Peltokorpi et al., focus on improvement of tactile imitation by the mother during play with her 3-year-old girl. A free play situation was compared with a play situation in which tactile imitation was guided. The authors concluded that, after intervention, tactile imitation as well as emotional availability increased. Wolthuis et al., demonstrated in an intervention study involving eight dyads of children and their communication partners that it was possible to describe and to monitor communication development over time with the help of the Layered Communication Model. Based on video-observations, it was shown that in particular the second layer (“joint attention” and “naming”), and the third layer (“symbolic communication” and “perspective taking”) proved to be useful in estimating the communication levels of the dyads.

Worm et al. studied the added value of multiparty conversations in three focus group sessions with 24 communication partners. The main characteristics of multiparty conversations was described: a minimum of three people involved in the conversation with at least one who has congenital deafblindness. The participants emphasized that multiparty conversations needed to be encouraged more. Partner competences such as positive beliefs, preparation of the multiple party conversations, repetitions and low communication speed were considered important.

Two studies in this Research Topic pay attention to learning, one that addresses cognitive abilities and one tactual perception. Nicholas reviews different evaluation approaches for reliable cognitive assessments. The assessors should be aware of the limitations of norm-references tests. Multiple assessment approaches are addressed such as: multi-method, multi-informant assessment, ecological assessment and dynamic assessment. The use of multiple assessment approaches is recommended as necessary to reveal the genuine cognitive abilities of these children.

Costain hypothesizes in a theoretical article, that touch hypersensitivity, also known in educational literature as tactile aversion, tactile shyness or defensiveness, can be considered as “overwhelming subjectivity” in the tactual perception. For partners of people with congenital deafblindness it is an important goal to be aware of the “overwhelming activation of tactual subjectivity.”

Two studies focus on access of environmental information during a visit to a cathedral and were part from a collaborative cross-linguistic research project between researchers from Norway and Sweden. Gabarró-López and Mesch aimed to contribute to the field of tactile sign language interpreting by describing how interpreters were able to convey environmental information to two older women with deafblindness. Video-observations were annotated with ELAN in several categories. A variety of strategies were used, including Swedish Sign Language, using locative points to show locations with some type of contact with the body of the deafblind individuals, depicting shapes on the palm of the hand, using objects to depict shapes, and touching elements of the cathedral with the hands or with the feet such as surfaces and walking around. The researchers did not observe any use of haptic signs to convey environmental information, and they recommend further research on this strategy. The study by Raanes addressed the research question: What elements are involved in descriptions to provide deafblind individuals access to their environments? Raanes analyzed extracts of data from four deafblind informants (three women and one man) with a mean age of 59 and their eight interpreters. Findings are based partly on video transcriptions made with ELAN and partly on conversation analysis. Four strategies were identified: (1) the repertoire of multimodal communicative tools; (2) topicalizations; (3) dialogical approach and (4) the impact of space and bodily orientation. This study has implications for a broader understanding of the repertoire of multimodal interaction and how such interaction may be used as inputs in the communication processes.

## Importance of Assistive Technology

Even though assistive technology plays an important part in most contributions to this Research Topic, the articles in this chapter specifically explored the importance of assistive technology for persons living with deafblindness in the context of Social Participation, Communication, as well as Orientation and Mobility.

Jaiswal et al. conducted individual interviews with 16 older adults living with age-related dual sensory impairment, exploring how they experience barriers and facilitators to social participation. While the availability of social support and the use of, and access to, assistive devices facilitated their social activities, the inaccessibility of the built environment and the cost and limited availability of accessible transportation made participation more difficult.

These findings were echoed in the results of a systematic literature review by Dyzel et al., where the authors examined what is currently known about the ability of assistive technology to promote communication and social interaction for people living with deafblindness. Using Technology Readiness Levels, the authors found that the technical maturity of the assistive technology currently available for persons with deafblindness still remains very much at the developmental and evaluative stage, and that most technologies are not yet freely available to the public. Focusing specifically on way-finding apps, Parker et al. asked nine adults living with deafblindness about their lived experiences when using personal mobile phones and wayfinding apps during independent travel in urban environments. The results clearly demonstrate the need for equity and inclusion in the design of assistive technology, and highlight the design limitations that still need to be overcome.

## Research, Professionalization, and Support

Studies in this chapter focus on a variety of themes related to research, professionalization and support. Damen et al. focus on research, by comparing Social Validity Ratings of 25 caregivers on perceived relevance, feasibility and effectiveness of the High-Quality Communication intervention, along with the observed effects of that same intervention in people with congenital deafblindness. No associations were found between the observed effectiveness of the intervention and the caregivers' opinions on the different aspects, except on feasibility which suggests that the perceived success of the intervention was influenced by caregivers' experienced competency in supporting the communication. It was recommended that multiple data sources for social validity assessment be used in future research.

Hartmann chose a case-study method of inquiry regarding team collaboration between school professionals. The study focused on professional learning of five professionals on one team educating a first-grade student with deafblindness who was taught in a general education classroom. The findings suggest that professional collaborating and learning was “difficult to grasp” and that for professional learning it is important that teachers are open to “education of all children.”

The very unique study of Prause et al. focuses on Existential Care for persons with age-related deafblindness. Open narrative interviews were conducted with five chaplains of the Norwegian Deaf Church with the aim to explore participants' lived experiences with providing existential care. Establishing trust and confidentiality appears to be a prerequisite. The participants emphasized the importance of acknowledging their negative feelings and addressing new perspectives on life with deafblindness. Existential care can be provided by: using oneself, building a safe foundation, alleviating burdens, and creating inclusive fellowships. Creating fellowships was not easy because of the diversity in communication and in personal needs.

The study by Dunsmore et al. aimed to explore the social experiences of family carers for older adults with a Dual Sensory Impairment. In their study, eight interviews with carers were analyzed according the Grounded Theory Methodology. Consistent with and adding to the earlier described study of Wahlqvist, Bjork et al., the key factors of social isolation, social effort, and negotiating relationships were found to often have a negative impact on social relations and the care relationship itself. This means that support of persons with deafblindness needs to include the family carers' perspective.

We hope that this Research Topic will help to inspire many more studies in the field of deafblindness and will cross-inspire researchers from the three subgroups of congenital, acquired, and age-related deafblindness. This collection of articles will facilitate collaboration and interdisciplinary interaction to maximize the visibility of deafblindness and move its research agenda forward. It is clear that much more research is needed, specifically in ways that can unite overlapping priorities of the three subgroups of individuals living with congenital, acquired or age-related deafblindness. The gaps that emerge, when taking a larger perspective on this Research Topic, indicate that we need to increase the capacity for training experts in deafblindness that have an interdisciplinary expertise. Such training will result in increased collaboration among experts, improve the training of specialized teachers, and the development of better strategies to improve the quality of life for individuals who live with a combined vision and hearing impairment. Currently, a larger multi-volume book proposal is under review to summarize the current state of knowledge on deafblindness and life-long learning, with the goal of forming a foundation for the field of deafblindness to build a strong evidence-based practice approach. This approach will benefit individuals living with deafblindness of all ages and all levels of sensory impairment severity, and will improve assessment and intervention options in education and rehabilitation for all people with a dual sensory loss.

## Author Contributions

All authors listed have made a substantial, direct, and intellectual contribution to the work and approved it for publication.

## Conflict of Interest

The authors declare that the research was conducted in the absence of any commercial or financial relationships that could be construed as a potential conflict of interest.

## Publisher's Note

All claims expressed in this article are solely those of the authors and do not necessarily represent those of their affiliated organizations, or those of the publisher, the editors and the reviewers. Any product that may be evaluated in this article, or claim that may be made by its manufacturer, is not guaranteed or endorsed by the publisher.
